# Waste Symbiosis through the Synthesis of Highly Crystalline LTA and SOD Zeolites

**DOI:** 10.3390/ma17174310

**Published:** 2024-08-30

**Authors:** Magali Teresinha Ritter, Isabel Padilla, María Ángeles Lobo-Recio, Maximina Romero, Aurora López-Delgado

**Affiliations:** 1 Department of Materials, Eduardo Torroja Institute for Construction Sciences (IETcc-CSIC), Serrano Galvache Street, 4, 28033 Madrid, Spain; magali.ritter@posgrad.ufsc.br (M.T.R.); isabel.padilla@ietcc.csic.es (I.P.); mromero@ietcc.csic.es (M.R.); 2 Department of Environmental Engineering, Federal University of Santa Catarina (UFSC), Campus Reitor João David Ferreira Lima, Florianópolis 88040-900, SC, Brazil; maria.lobo@ufsc.br; 3 Department of Energy and Sustainability, Federal University of Santa Catarina (UFSC), Campus Araranguá, Rodovia Governador Jorge Lacerda, 3201, Jardim das Avenidas, Araranguá 88906-072, SC, Brazil

**Keywords:** sol–gel synthesis, waste-based zeolites, LTA and SOD zeolites, hazardous waste, salt slag, rice husk ash

## Abstract

In recent years, the demand for natural and synthetic zeolites has surged due to their distinctive properties and myriad industrial applications. This research aims to synthesise crystalline zeolites by co-recycling two industrial wastes: salt slag (SS) and rice husk ash (RHA). Salt slag, a problematic by-product of secondary aluminium smelting, is classified as hazardous waste due to its reactive and leachable nature, though it is rich in aluminium. Conversely, RHA, an abundant and cost-effective by-product of the agro-food sector, boasts a high silicon content. These wastes were utilised as aluminium and silicon sources for synthesising various zeolites. This study examined the effects of temperature, ageing time, and sodium concentration on the formation of different zeolite phases and their crystallinity. Results indicated that increased Na^+^ concentration favoured sodalite (SOD) zeolite formation, whereas Linde type–A (LTA) zeolite formation was promoted at higher temperatures and extended ageing times. The formation range of the different zeolites was defined and supported by crystallographic, microstructural, and morphological analyses. Additionally, the thermal behaviour of the zeolites was investigated. This work underscores the potential to transform industrial waste, including hazardous materials like salt slag, into sustainable, high-value materials, fostering efficient waste co-recycling and promoting clean, sustainable industrial production through cross-sectoral industrial symbiosis.

## 1. Introduction

Demand for zeolites has grown considerably in recent years, driven mainly by the detergent industry, where synthetic zeolites have been used to replace phosphate-based agents, which are highly polluting to the environment. In addition to detergents, zeolites have been widely applied in several other areas, including petrochemicals, biotechnology, fertilisers, construction, gas separation, environmental remediation, and even biodiesel production [1]. 

A recent analysis by Markets and Markets [2] predicts a 3.1% Compound Annual Growth Rate (CAGR) in the global zeolite market from 2021 to 2026, expanding from 4872 metric tonnes to 5453 metric tonnes, with synthetic zeolites comprising approximately 42% of the total. Despite the availability of natural zeolites, they are generally contaminated to varying degrees by other minerals, metals, or other zeolites, making them unsuitable for commercial and industrial applications that require high purity and uniformity [3]. Consequently, there is growing interest in the synthesis of zeolites, which allows the production of zeolitic structures with high purity, more uniform sizes, better ion exchange capacity, high selectivity, and higher thermal resistance [4].

**Figure 1:**
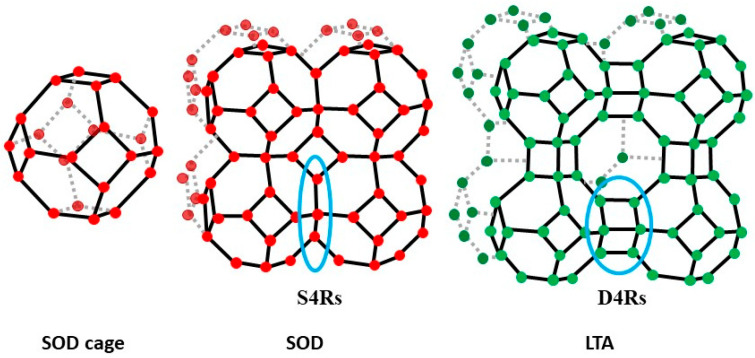
LTA- and SOD–type frameworks. The double D4R and single S4R bonds are in blue.

With regard to synthetic zeolites, the LTA type–A (LTA) zeolite is one of the most widely used, especially due to its three-dimensional structural arrangement, which provides high adsorption and ion exchange capacity. This makes it suitable for use as molecular sieves and adsorbents in cooling, cleansing, and water softening systems [1,4]. The LTA zeolite (Na_12_Al_12_Si_12_O_48_·27H_2_O) framework is formed by the so-called β-cages or sodalite cages (24 T atoms [T = Si^4+^ or Al^3+^]), in which the primary units are SiO_4_ and AlO_4_ tetrahedra (Figure 1). These units are connected to the six nearest neighbouring β-cages by double T4 rings [D4Rs]. When connected to neighbouring β-cages via single T4 rings [S4Rs], the sodalite zeolite structure is formed, which is the simplest structure among zeolites [5]. The cubic framework of the sodalite (SOD) zeolite (Na_8_Al_6_Si_6_O_24_(OH)_2_·2H_2_O) has small pore sizes and thus a low application potential for ion exchange and molecular sieving. However, it is considered a promising material as a membrane separator for small molecules of liquids and gases, including H_2_ and He [6]. 

The formation of LTA or SOD zeolites is defined by the ideal synthesis condition, as the development of the desired zeolite phase depends on a specific temperature range, reaction time, and the molar ratio of the initial gel composition, which directly affect the nucleation and crystal growth processes [7].

In recent years, the synthesis of zeolites using secondary raw materials has attracted increasing attention in order to reduce production costs and mitigate the associated environmental impacts. In this sense, a great variety of wastes have been tested to produce LTA and SOD zeolites, including waste glass [8,9,10], rice husks [11,12,13,14,15], fly ash [16,17,18,19,20,21,22,23,24,25,26], alum sludge [27,28,29,30], and aluminium waste [6,31,32,33,34,35,36]. The synthesis methods applied often involve several steps and high temperature or long reaction time. Melo et al. [12] reported the synthesis of LTA through a hydrothermal process, employing a commercial sodium aluminate solution and a sodium silicate derived from RHA treated at 1200 °C for 2 h. Abdelharam et al. [32] employed a sol–gel process involving RHA and aluminium can waste at 150 °C for 12 h. RHA was also used by Simanjuntak et al. [13] together with food-grade aluminium foil in a process that entailed the alkaline dissolution of both wastes, followed by the ageing of the solution mixture for 24 h at room temperature and a crystallisation stage at 100 °C for 72 h; the resulting product was then subjected to a calcination process at 550 °C for 8 h to form zeolite A.

Among aluminium waste, salt slag is the main waste produced by the secondary aluminium industry, generating approximately 0.5 tons of salt slag for every ton of recycled aluminium produced [37]. Based on data published by Statista (2023) [38], it is estimated that more than 13 million metric tons of salt slag will be generated worldwide by 2027. This figure has almost tripled in 20 years and is on an upward trend due to the increased use and recycling of aluminium [39]. In addition to the large volumes, the management and disposal of this waste is a major concern due to its potential for environmental contamination. Salt slag is considered toxic and hazardous waste, highly harmful, flammable, irritating, and leachable, according to the European Catalogue of Hazardous Waste [40] code 10 03 08. Its irritant properties pose a threat to human health and can cause damage through contact with the skin or mucous membranes, ingestion, and inhalation. Disposal in landfills is an environmental catastrophe due to the leachable salts, which can cause irreversible impacts on groundwater and soil [41]. Furthermore, the high reactivity of salt slag in contact with water leads to the formation of toxic gases (NH_3_, CH_4_, H_2_S, H_2_, and PH_3_), which also pollute the atmosphere [37]. Nevertheless, its aluminium-rich composition makes salt slag a potential candidate for producing zeolites.

Although, as mentioned above, some authors have used different aluminium wastes as precursor materials in the synthesis of zeolites, few studies have been conducted with salt slag [37,42]. In addition, commercial silicate solutions were used as the source of silicon in these works.

Concerning silicon waste, rice husk ash (RHA) has been identified as a promising low-cost alternative to commercial silica [43]. It is a silicon-rich material resulting from the thermal transformation of rice husks and is considered to be one of the most abundant agro-food waste products. According to the Food and Agriculture Organization of the United Nations [44], more than 31 million tons of RHA were generated worldwide in 2023. Although rice husk ash is not hazardous waste as salt slag is, its high production, slow biodegradation, small particle size, and need to be disposed of in landfills cause several environmental problems. 

In a previous paper, the authors studied the synthesis of NaP-type zeolite from salt slag and RHA by a hydrothermal method. Moreover, a central composite rotational design (CCRD) was applied to determine the best experimental conditions required to obtain NaP zeolite [45]. This work reports for the first time the synthesis of LTA and SOD zeolites from two unconventional raw materials, such as hazardous aluminium salt slag and rice husk ash. The aim of this study was to promote the co-recycling of these two wastes via the sol–gel process as a way of minimising the environmental impacts associated with their management. The evolution in terms of crystallinity, microstructure, and morphology of zeolitic materials synthesised under different experimental conditions was evaluated, assessing the influence of reaction time, temperature, and alkalinity. In addition, the thermal behaviour of the zeolites and their cation exchange capacity were also studied. The novelty and applicability of this work fall under the development of a synthesis method under mild conditions that makes it possible to produce highly crystalline LTA- and SOD-type zeolites from the combination of two industrial wastes.

## 2. Materials and Methods

### 2.1. Raw Materials

For the synthesis of zeolites, two different industrial wastes were employed: a hazardous waste from the secondary aluminium industry named salt slag (SS) and a waste from the agro-food industry, specifically rice husk ash (RHA). The wastes were selected for their respective contents of alumina (SS) and silicon (RHA), the two main components of zeolite composition. SS was supplied by Alusigma S.A. (Gijón, Spain), and its chemical composition mainly consists of Al_2_O_3_ (63.5 wt.%) and smaller amounts of MgO (7.9 wt.%), SiO_2_ (7.7 wt.%), CaO (4.5 wt.%), and Fe_2_O_3_ (3.0 wt.%). Rice husk ash (RHA) was used to provide the necessary amount of silicon for zeolite synthesis. The RHA sample was supplied by Herba Ricemills S.L.U. (Seville, Spain). The main component of RHA is SiO_2_ (89.7 wt.%), followed by minor amounts of K_2_O (3.6 wt.%), P_2_O_5_ (1.7 wt.%), and CaO (1.3 wt.%). 

The complete characterisation of both wastes (SS and RHA) was reported in a previous work [45]. Furthermore, a commercial sample of LTA zeolite, used for comparative purposes, was supplied by Industrias Químicas del Ebro, S.A. (Zaragoza, Spain).

### 2.2. Zeolite Synthesis

The waste-based zeolites were synthesised using a sol–gel process followed by an ageing step. Firstly, aluminate and silicate solutions were prepared by treating the initial SS and RHA in an alkaline medium (NaOH solution). Preliminary studies were conducted at different times (1–24 h), temperatures (room temperature—120 °C), and alkalinities (1–5 M) in order to determine the best conditions for obtaining aluminate and silicate solutions with the highest aluminium and silicon contents, respectively. The highest Al concentration, 19.88 g/L, was achieved by treating 0.15 g/mL of SS with a 5 M NaOH solution for 1 h at 100 °C. Similarly, the highest silicon content (58.34 g/L) of the silicate solution prepared from RHA was achieved by treating 0.16 g/mL of RHA with a 3 M NaOH solution for 3 h at 120 °C. The Na concentrations in the aluminate and silicate solutions were 105.3 and 69.66 g/L, respectively.

The sol–gel synthesis was performed by adding the silicate solution, in the required amounts to obtain a Si/Al ratio = 1, to the aluminate solution at room temperature with constant stirring. The resulting gel was kept under constant stirring under different ageing conditions (Table 1). Due to the high sodium content of the aluminate and silicate solutions, only distilled water in the appropriate volumes was added to the synthesis, and no additional NaOH solution was required. The different Na^+^ concentrations used focused on obtaining the LTA zeolite phase, while the temperatures and ageing times tested aimed to increase the crystallinity of the resulting samples [5,46]. Nine experiments were carried out under different ageing conditions to evaluate their effect on the type and properties of the zeolites obtained. The samples obtained were labelled Z1–Z9 (Table 1).

After the tests, the resulting solid products were filtered, washed with distilled water, and dried at 100 °C for 24 h. The samples were then characterised by XRD, SEM, and FTIR according to the procedures described in the following Section 2.3. In addition, their cation exchange capacity (CEC) and thermal behaviour (TG/DTA) were also determined.

### 2.3. Characterisation Techniques

The composition of the aluminate and silicate solutions extracted from salt slag and RHA, respectively, was analysed using an inductively coupled plasma optical emission spectrometer, ICP-OES (Varian 725-ES, Agilent Technology, Santa Clara, CA, USA). The mineralogical characterisation of the zeolites was carried out by X-ray diffraction (XRD) using a Bruker D8 Advance diffractometer (Bruker, Champs–sur–Marne, France) with CuKα radiation, 2θ from 5° to 60°, and a scan rate of 2θ of 0.02°, 5 s per step. Diffrac.Suite EVA Plus 13.0 software (Bruker, AXS GmbH, Karlsruhe, Germany) was used to semi-quantify the crystalline phases of the zeolitic materials obtained. A crystallographic study of the zeolites was performed, which included the determination of interplanar spacing and network parameters. The interplanar spacing d (Å) was calculated by applying Bragg’s law (Equation (1)), where n is a natural number other than zero (n = 1), λ is the wavelength of the incident radiation (0.154 nm), and θ is the diffraction angle.

(1) d=nλ/2sinθ

The lattice parameter a was calculated according to the crystalline system (cubic) using Equation (2), where d corresponds to the interplanar spacing and hkl to the Müller indices relative to the diffraction planes.

(2) $$a=d\sqrt{\left(h^{2}+k^{2}+l^{2}\right)}$$

Thermogravimetric and differential thermal analysis (TG-DTA) was carried out on a Thermoanalyzer model SDT-Q600 (TA Instruments, New Castle, DE, USA), under an air flow of 100 mL/min and a heating rate of 10 °C/min. The Fourier transform infrared (FTIR) spectra (Nicolet Nexus 670–870, Nexus, Singapur, Malasia) were recorded on KBr discs in the 400–4000 cm^–1^ range. The cation exchange capacity (CEC) of the zeolites was determined by the ammonium ion exchange method using an NH_4_Cl solution (1 M), as described in the Standard number NC 626 [47].

## 3. Results and Discussion

### 3.1. Effect of Ageing Time, Temperature, and Alkali Concentration

**Figure 2:**
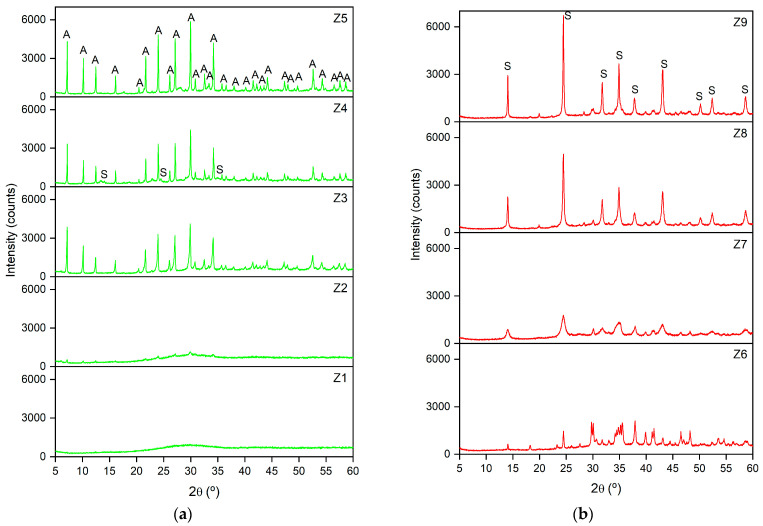
XRD patterns of the waste-based materials (**a**) Z1 to Z5 (green) and (**b**) Z6 to Z9 (red), synthesised under different experimental conditions [A = LTA zeolite (PDF 73–2340) and S = SOD zeolite (PDF 76–1639)].

The XRD patterns of the nine samples of the zeolites synthesised using aluminate and silicate solutions from SS and RHA are shown in Figure 2a (samples Z1 to Z5) and Figure 2b (samples Z6 to Z9), based on the predominant zeolitic material obtained, for better viewing. The crystallographic parameters, including the intensity, diffraction angle (2θ), and Full Width at Half Maximum (FWHM) of the most intense reflections, are shown in Table 2. In addition, the semi-quantification of the most crystalline phases identified using Diffrac.Suite EVA software is presented, as is the crystallite size (D) determined from the most intense peak of the zeolite phase using the Scherrer equation: D = (0.9·λ)/(FWHM·cos θ), where λ is the X-ray wavelength (0.154 nm) and θ is the diffraction angle (in rad).

From Figure 2 and Table 2, it can be observed that, with the exception of sample Z1, in which no crystalline phase could be identified, and sample Z2, with an incipient appearance of peaks, all the other samples resulted in the formation of crystalline zeolites. By increasing the ageing time from 24 h (Z1) to 240 h (Z2), the XRD pattern shows the development of small peaks with a profile characteristic of the cubic zeolite LTA, with a crystallite size of 22 nm. Increasing the Na^+^ concentration from 1.27 mol/L (Z2) to 2.36 mol/L (Z3) at 240 h and room temperature resulted in the development of narrow, very intense, and well-defined peaks (>4000 counts), which fit well with those of the XRD pattern of the LTA zeolite from the International Centre for Diffraction Data (ICDD), reference file PDF 73-2340. A crystallite size of 45 nm was calculated for this sample. This result highlights that a higher concentration of Na^+^ promotes the formation of LTA when the ageing time is extended. Both samples Z4 and Z5, synthesised with a NaOH concentration of 1.27 mol/L at 70 °C, resulted in 100% zeolitic material. However, for the sample synthesised over 15 h (Z4), several peaks corresponding to the SOD–type zeolite were observed along with corresponding ones to LTA, the latter with a crystallite size of 55 nm. By increasing the ageing time to 24 h (Z5), a single LTA zeolite phase was obtained with very intense and well-defined peaks (>5800 counts) and a crystallite size of 53 nm. This indicates that a longer ageing time favours the formation of LTA zeolite.

Regarding samples Z6, Z7, Z8, and Z9, the XRD patterns (Figure 2b) principally showed peaks corresponding to the SOD–type zeolite (ICDD PDF 76-1639). A certain amorphous phase content, decreasing from Z6 (close to 40%) to Z9 (around 25%), is also consistent with the background of the patterns. At room temperature, 240 h, and 4.12 mol/L of Na^+^ (Z6), SOD showed a peak intensity >1400 counts and a crystallite size of 45 nm. Increasing the temperature to 50 °C for 24 h while maintaining a Na^+^ concentration of 1.27 mol/L (Z7) resulted in SOD with a peak intensity >1700 counts and a crystallite size of 10 nm. At 70 °C for 6 h (Z8), the percentage of crystalline SOD in the sample reached 81.3%, with a maximum peak intensity around 5000 counts and a crystallite size of 30 nm. Finally, sample Z9, synthesised at room temperature for 120 h with 2.36 mol/L of Na^+^, resulted in the highest percentage of crystalline SOD and the highest peak intensity (>6700 counts), with a crystallite size of 41 nm.

**Figure 3:**
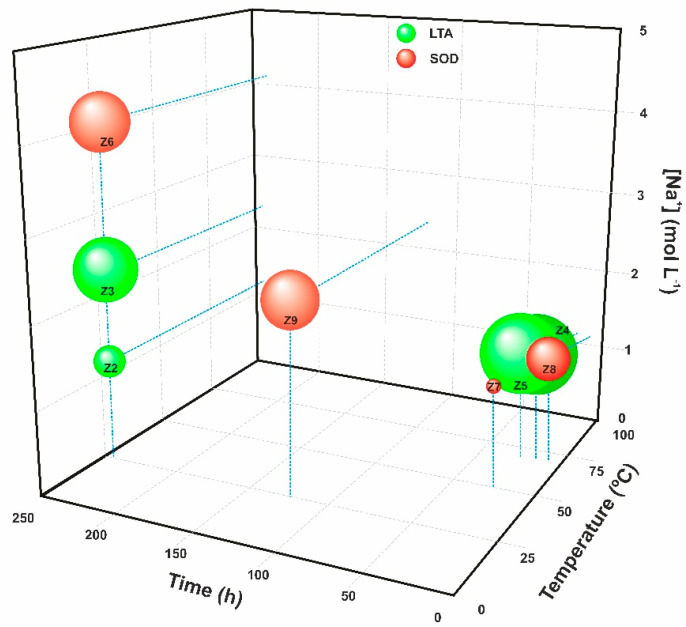
Zeolite phase and corresponding crystallite size obtained according to different experimental conditions (LTA in green and SOD in red).

The crystallite sizes of the different zeolite phases synthesised as a function of the experimental conditions applied (ageing time, temperature, and Na^+^ concentration) are shown in Figure 3. 

Several factors, including temperature, ageing, pressure, reagent sources, Si/Al ratio, and water content [46], influence not only the development of specific zeolite phases and their crystallinity but also the size of the crystals formed. Thus, increasing the ageing time from 24 to 240 h at room temperature resulted in the evolution from a geopolymer (Z1) to the incipient formation of LTA zeolite (Z2). However, this trend was not observed at a higher temperature; in the synthesis conducted at 70 °C, extending the reaction time from 15 to 24 h resulted in similar crystallite sizes (samples Z4 and Z5). Sodium concentration had the most significant effect on crystallite size. Increasing the Na^+^ concentration from 1.27 mol/L to 2.36 mol/L caused the crystallite size of the LTA zeolites to increase from 22 nm (Z2) to 45 nm (Z3). Nevertheless, obtaining a specific zeolite phase is defined by the alkali concentration in the mixing reaction and the crystallisation kinetics [36,48]. Comparing samples Z2, Z3, and Z6, synthesised under the same conditions of time (240 h) and temperature (RT), revealed that increasing the sodium content resulted in the development of the SOD phase, known for its higher Na_2_O/Al_2_O_3_ ratio (close to 1.33 for the stoichiometric phase) compared to the LTA zeolite (Na_2_O/Al_2_O_3_ = 1). This conclusion is supported by the fact that a high concentration of NaOH solution (>3.5 M) destabilises the structure of the LTA zeolite, causing the destruction of double T4 rings [D4Rs] and leading to the binding of β-cages via single T4 rings [S4Rs] and the consequent formation of sodalite [5,46].

According to the results, longer ageing times and higher temperatures led to the formation of LTA–type zeolites. This behaviour was observed by comparing Z7 and Z5, when the temperature was increased from 50 to 70 °C to a fixed Na^+^ concentration of 1.27 mol/L, and with samples Z9 and Z3, which transitioned from an SOD zeolite to an LTA zeolite by doubling the ageing time (120 to 240 h). Shorter ageing times disproved this outcome when comparing samples Z8, Z4, and Z5, which transitioned from an SOD zeolite (6 h) to a mixture of LTA–SOD (15 h) and a pure LTA (24 h). This suggests that sodalite could serve as an intermediate phase that evolves into an LTA zeolite as the ageing time increases, as indicated by the mixture of zeolitic phases identified for an intermediate ageing time (Z4). Similar results were reported by other authors. Simanjuntak et al. [13], who synthesised zeolites using RHA and aluminium foil as raw materials, corroborate the findings, also reporting that an SOD zeolite was obtained with a shorter reaction time (48 h) compared to a longer time (72 h) which resulted in an LTA–type zeolite. This result suggests that an increase in reaction temperature enhances the partial dissolution of silica and alumina components from the gel into the aqueous phase and subsequently promotes the formation of crystal nuclei within the gel matrix [36]. It can be inferred that different heating rates lead to the formation of slightly different initial gels and consequently to the development of different zeolite phases. Thus, the interplay between temperature and ageing time is key for obtaining highly crystalline single-phase LTA zeolites.

In regard to the crystallite size, the values obtained are quite similar to those reported by other authors who have synthesised LTA– and SOD– type zeolites from wastes. Al-Dahri et al. [16] obtained LTA zeolite with a crystallite size of 45 nm from coal fly ash using a microwave-assisted method. The sol–gel synthesis performed by Asefa & Feyisa [49] from aluminium foil waste and sugarcane bagasse ash resulted in LTA zeolite with a crystallite size of 49 nm. Meanwhile, the SOD zeolite produced by this same method had crystallites ranging from 46 to 64 nm when aluminium can waste was used [50]. 

**Figure 4:**
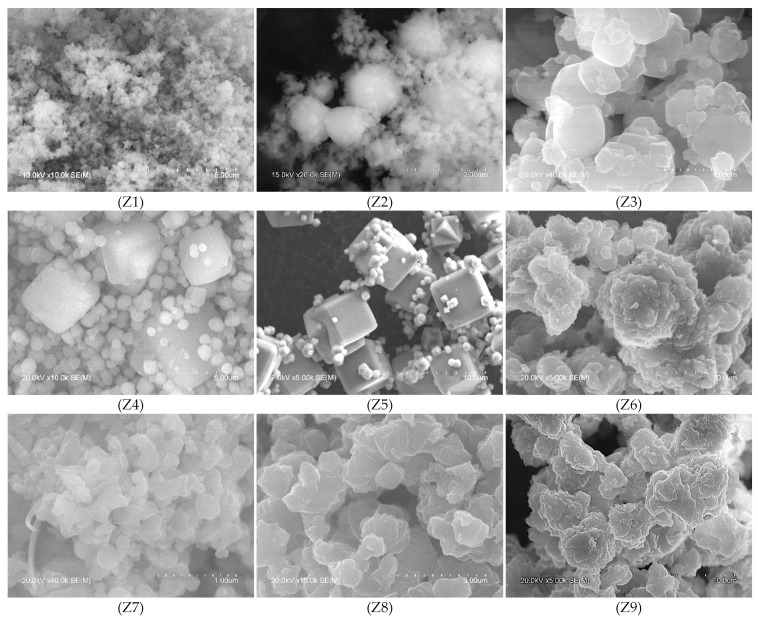
SEM images of the waste–based synthesised materials Z1–Z9.

The reported differences in crystallinity and structure were also observed in the morphology of the waste-based zeolites obtained under different ageing conditions (Figure 4). Corroborating the XRD analysis, the SEM micrograph of sample Z1 (Figure 4Z1) shows agglomerates of tiny, rounded particles (<0.1 µm) characteristic of geopolymeric materials, whereas in the case of sample Z2 (Figure 4Z2), larger particles (1–1.6 μm) have begun to develop which, although predominantly amorphous, present an incipient cubic morphology (1–1.6 μm) but without well-defined edges and boundaries. The micrograph of sample Z3 (Figure 4Z3) shows a stacking of particles with a cubic tendency and edges with a higher degree of definition than those observed in Z2, with sizes varying between 0.1 and 0.9 μm, indicating the formation of a more crystalline LTA zeolite. The lack of definition on the edges of the cubes is due to the low temperature during the ageing stage. The synthesis temperature affects the morphology of the zeolites, with low temperatures leading to the formation of rounder crystals and higher temperatures leading to more cubic shapes [1,51]. This is corroborated by the predominant presence of well-defined cubic crystals in the SEM images of samples Z4 (Figure 4Z4) and Z5 (Figure 4Z5), characteristics of crystalline LTA zeolite. In the first sample, the largest cubes ranged in size from 2.5 to 5 μm and the smallest from 0.2 to 1.5 μm. As for sample Z5, the cubic crystals exhibit perfectly defined and slightly chamfered edges. In this sample, some cubic twinned crystals are also observed, along with very small cubes (500 to 900 nm) developed on top of the larger ones. 

The morphology of the Z6–Z9 samples (Figure 4Z6–Z9) confirms the XRD analysis by presenting structures consistent with sodalite–type zeolites, similar to those reported by other authors [50,52]. SEM images of samples Z6 and Z9 show clusters (30–100 μm) of slightly rounded “flower-like” particles with average diameters of 2–10 μm, which are characteristic of the SOD–type structure [22]. 

### 3.2. Study of LTA and SOD Zeolites

As mentioned above, samples Z5 and Z9 correspond to well-defined LTA– and SOD–type zeolites, respectively, so both samples were subjected to more in-depth analysis.

**Figure 5:**
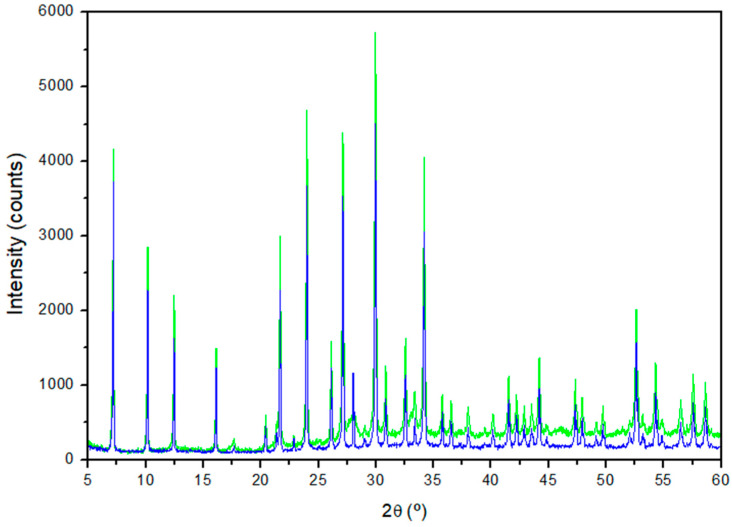
XRD patterns of the waste-based LTA zeolite (Z5) in green and commercial LTA zeolite (ZCOM) in blue.

Due to the high crystallinity and well-defined peaks of LTA zeolite obtained from Z5, its XRD pattern and crystallographic parameters were compared with those of a commercial LTA zeolite (ZCOM) (Figure 5, Table 3).

The LTA zeolite synthesised from SS and RHA showed an XRD profile quite similar to that of the commercial zeolite, with well-developed peaks and slightly higher intensities (Figure 5). The most significant peaks of the Z5 sample compared to the commercial LTA zeolite; the reference file PDF 73–2340 showed the similarity of the interplanar spacing values obtained as well as the relative intensities (I/I_0_) [53]. The most intense reflection of the synthesised LTA zeolite, centred at 29.96 ° (2θ), corresponds to the diffraction hkl index [6 4 4], according to PDF 73–2340. In addition, the lattice parameter a, calculated according to Equation (2), which considers the cubic crystal system of the LTA zeolite, was 12.29 Å, very similar to the 12.32 Å reported in the reference ICDD files.

In the case of the SOD zeolite, the most significant peaks of the Z9 sample coincide completely with the reference file PDF 76–1639 [54] (Table 4). The interplanar spacing and the relative intensities of the most intense reflections show practically identical values. The most intense reflection corresponding to the SOD phase was centred at 24.47° (2θ), which corresponds to the hkl index [2 1 1].

The calculated lattice parameter, which, as for the LTA zeolite, also considers the cubic-type crystal system of the SOD zeolite, was 8.90 Å, compared to 8.89 Å assigned by the reference file.

**Figure 6:**
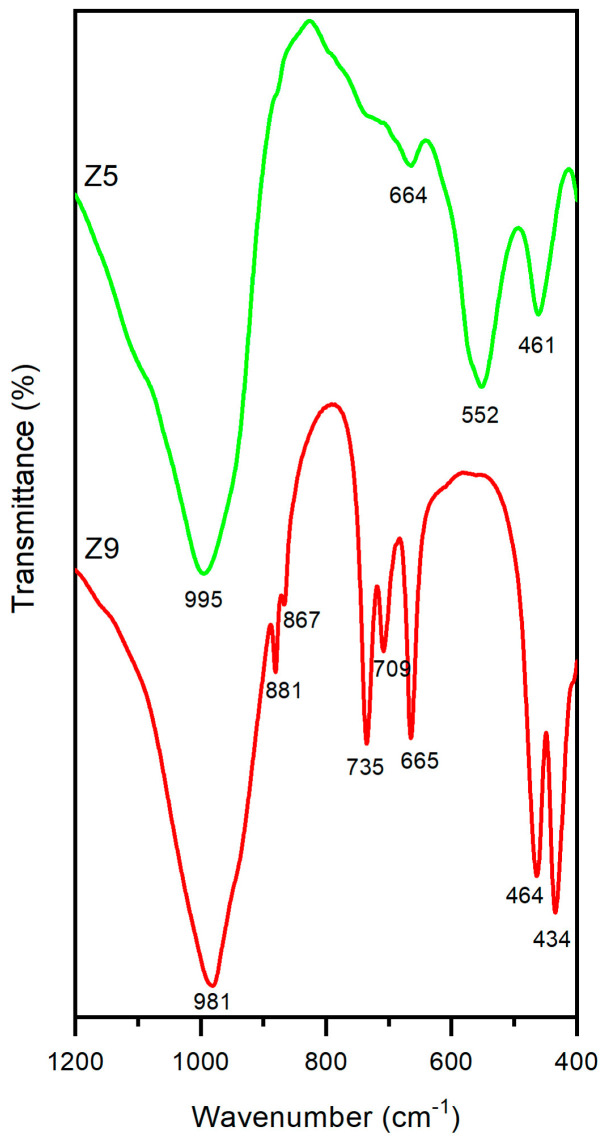
FTIR spectra of LTA (Z5) in green and SOD (Z9) in red.

The FTIR spectra of samples Z5 (LTA) and Z9 (SOD) (Figure 6) were recorded in the mid-infrared wavenumber region (1200 to 400 cm^−1^), where the fundamental vibrations of the framework (Si, Al)O_4_ tetrahedra are located [55]. The spectrum of sample Z5 shows the four absorption bands characteristic of LTA zeolite. The bands at 995 and 664 cm^−1^ are due to asymmetrical and symmetrical internal stretching vibrations, respectively. The band at 461 cm^−1^ corresponds to the Si-O-Al bending mode, and the medium-intensity vibration at 552 cm^−1^ is attributed to the vibration of the secondary structural units [D4Rs] [36]. Similar FTIR values were reported by López-Delgado et al. [31], who also prepared LTA zeolite from an aluminium waste, as well as the those reported by Vegere et al. [56] for zeolite 4A prepared from commercial raw materials. Thispoints the high purity of the synthesised waste-based LTA zeolite. Sample Z9 showed the typical triplet of SOD zeolite, with bands at 735, 709 and 665 cm^−1^ corresponding to the symmetrical stretching mode. The two bands at 464 and 434 cm^−1^ represent the octahedral bending mode. In addition, two low-intensity bands are observed at 881 and 867 cm^−1^, attributable to the symmetrical external stretching of T-O-T (T = Si and/or Al) [32]. These results are corroborated by Sánchez-Hernández et al. [6] for SOD zeolite produced from an aluminium waste and commercial sodium silicate.

**Figure 7:**
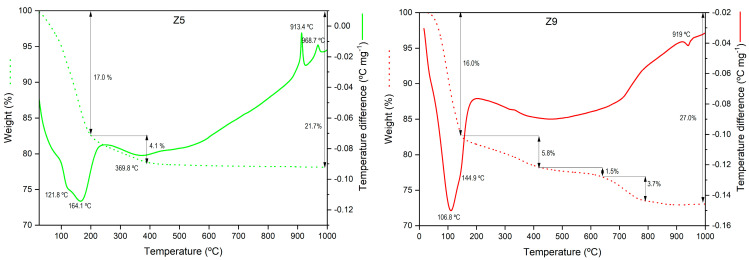
TG (dot line) and DTA (solid line) for the selected waste-based zeolites LTA (Z5) and SOD (Z9).

The TG-DTA curves of samples Z5 (LTA) and Z9 (SOD) are shown in Figure 7. Both samples exhibit endothermic effects below 250 °C. The structured profile of these bands indicates that the dehydration of both zeolites occurs in several overlapped steps and is due to different types of water (absorbed, zeolitic, etc.). Concerning LTA, a second mass loss takes place below 400 °C and is associated with the loss of water due to a dehydroxylation process. The total mass loss for sample Z5, according to the TG curve, was 21.7%, similar to the loss observed for a commercial zeolite [57]. This value corresponds to a loss of approximately 26 water molecules, which is quite similar to the corresponding one for the stoichiometric theoretical LTA zeolite (Na_12_Al_12_Si_12_O_48_·27H_2_O). After that, no mass loss is observed in the TG curve, but the DTA curve exhibits two exothermic peaks centred at 913 and 969 °C. These peaks are attributed to the topotactic transformation of the cubic framework of LTA zeolite into the hexagonal framework of nepheline (NaAlSiO_4_). Several authors reported that this transformation occurs at temperatures higher than 700 °C [58]. A temperature of 890 °C has also been reported for LTA zeolite obtained at pilot scale from an aluminium waste and commercial water glass [31]. Selvaraj et al. [59] also observed two exothermic peaks between 800 and 900 °C in the DTA curve of a commercial LTA zeolite due to the transformation and recrystallisation of nepheline.

In the case of sample Z9 (SOD), a total mass loss of 27% took place from room temperature up to 800 °C, corresponding to the release of 22 water molecules. Between 400 and 800 °C, the mass loss of 5.2% can be attributed to the crystallisation and structural water, which fit well to the theoretical value for a sodalite stoichiometry of Na_8_Al_6_Si_6_O_24_(OH)_2_·2H_2_O [60]. Above 790 °C, the DTA curve shows an inflection point without any corresponding mass loss. This observation suggests the onset of a gradual transformation from sodalite to nepheline, although the transformation remains incomplete at the test temperature.

Overall, the zeolites studied exhibit the ability to retain their structure and only lose water during thermal treatment at temperatures below 800 °C for both Z5 and Z9. This characteristic makes these zeolites useful in processes requiring high temperatures or those needing high-temperature treatment for regeneration [57].

Concerning the cation exchange capacity (CEC), the value for Z5 was 3.40 meq/g, higher than that of Z9 (1.82 meq/g). The CEC is one of the main requirements for the use of zeolites, especially in detergent formulation and water decontamination [61,62]. The CEC results found in this study are remarkable when compared to the values reported by other authors who synthesised A-type zeolites using different wastes [17,30,31,48,63]. Pangan et al. [48] reported a CEC value of 2.44 meq/g for an LTA zeolite synthesised from corn straw ash by a hydrothermal method at 90 °C for 9 h, while other authors reported values of CEC lower than 2 meq/g for zeolites obtained from aluminium slag milling waste [31], fly ash by a microwave-assisted hydrothermal process [17], or alum sludge by hydrothermal synthesis at 90 °C for 9 h [30]. The high CEC value of the LTA zeolite synthesised in this study suggests its promising application in processes such as the treatment of metal-contaminated wastewaters. The CEC of SOD zeolite, although lower than that of LTA, was higher than that reported by other authors [6].

## 4. Conclusions

The co-recycling of hazardous aluminium salt slag and rice husk ash through a synthesis process under mild conditions resulted in highly crystalline LTA and SOD zeolites. A highly crystalline LTA zeolite was obtained at a temperature of 70 °C for 24 h, while under the same conditions, the SOD zeolite was synthesised in only 6 h. The results showed the great influence of the experimental conditions on the development of a specific zeolite phase. The increase in sodium concentration favours the formation of SOD zeolite. The effect of temperature on the crystallinity of the zeolites is much more significant than the effect of ageing time. A high value of cation exchange capacity of 3.40 meq/g was obtained for the LTA. This indicates that the LTA-type zeolite prepared from wastes could have potential applications in the same way as commercial zeolites. The synthesis of zeolites can be considered as a new alternative route to conventional waste management methods, especially for hazardous wastes such as salt slag, leading to the production of value-added materials, which are in increasing demand worldwide and have significant applications in several fields. The valorisation of these wastes through their conversion into advanced materials, such as LTA- and SOD-type zeolites, contributes above all to saving natural resources and preserving the environment. Finally, yet importantly, this approach favours the circular economy, creating a symbiosis between the different industrial segments, given that many industries that generate aluminium and agro-food waste use zeolites in their industrial gas and effluent treatment systems.

## Figures and Tables

**Table 1:** Experimental ageing conditions of the synthesised waste-based materials and Na^+^ concentration in the solution.

Samples	T (°C)	t (h)	[Na^+^] (mol/L)
Z1	RT	24	1.27
Z2	RT	240	1.27
Z3	RT	240	2.36
Z4	70	15	1.27
Z5	70	24	1.27
Z6	RT	240	4.12
Z7	50	24	1.27
Z8	70	6	1.27
Z9	RT	120	2.36

RT: room temperature.

**Table 2:** Crystallographic parameters (intensity, 2θ, and FWHM), semi-quantification of the zeolite phases, and crystallite sizes (D) of the synthesised waste-based materials.

Samples	Phase	Zeolite (%)	Intensity (Counts)	2θ (°)	FWHM (°)	D (nm)
Z1	Amorphous phase	-	-	-	-	-
Z2	LTA + amorphous phase	47.4	1162	29.92	0.3743	22
Z3	LTA	100	4101	29.88	0.1848	45
Z4	LTA SOD	95.6 4.4	4423	29.96	0.1496	55
Z5	LTA	100	5864	29.96	0.1564	53
Z6	SOD	24.6	1464	24.49	0.1808	45
Z7	SOD	30.0	1783	24.48	0.7829	10
Z8	SOD	81.3	4955	24.48	0.2703	30
Z9	SOD	81.8	6723	24.47	0.1992	41

FWHM: Full Width at Half Maximum; D: crystallite size, determined from the most intense reflection.

**Table 3:** Crystallographic parameters of waste-based LTA zeolite, commercial LTA zeolite, and reference ICDD files.

Z5 (LTA)			ZCOM			PDF 73-2340			
d (Å)	2θ (°)	I/I_0_	d (Å)	2θ (°)	I/I_0_	d (Å)	2θ (°)	I/I_0_	hkl
12.27	7.20	73	12.27	7.20	64	12.31	7.18	69	[2 0 0]
8.69	10.17	51	8.69	10.17	39	8.70	10.16	46	[2 2 0]
7.09	12.47	40	7.09	12.47	28	7.10	12.45	51	[2 2 2]
4.10	21.68	54	4.10	21.68	39	4.10	21.65	39	[6 0 0]
3.71	24.00	82	3.71	24.00	63	3.71	23.97	54	[6 2 2]
3.41	26.12	29	3.41	26.12	21	3.41	26.09	8	[6 4 0]
3.29	27.13	77	3.29	27.13	60	3.29	27.09	67	[6 4 2]
2.98	29.96	100	2.98	29.96	100	2.98	29.92	100	[6 4 4]
2.75	32.56	30	2.75	32.56	19	2.75	32.52	22	[8 4 0]
2.62	34.20	72	2.62	34.18	52	2.62	34.15	50	[6 6 4]

**Table 4:** Crystallographic parameters of waste-based SOD zeolite.

Z9 (SOD)			PDF 76–1639			
d (Å)	2θ (°)	I/I_0_	d (Å)	2θ (°)	I/I_0_	hkl
6.30	14.05	44	6.29	14.08	44	[1 1 0]
3.64	24.47	100	3.63	24.51	100	[2 1 1]
2.81	31.79	37	2.81	31.81	40	[3 1 0]
2.57	34.88	55	2.57	34.93	48	[2 2 2]
2.10	43.10	49	2.10	43.14	56	[4 1 1]

## Data Availability

The raw data supporting the conclusions of this article will be made available by the authors on request.
